# Medicinal Plants in the Treatment of Hypertension: A Review

**DOI:** 10.34172/apb.2021.090

**Published:** 2020-11-01

**Authors:** Raha Kamyab, Hossein Namdar, Mohammadali Torbati, Morteza Ghojazadeh, Mostafa Araj-Khodaei, Seyyed Mohammad Bagher Fazljou

**Affiliations:** ^1^Department of Persian Medicine, Faculty of Traditional Medicine, Tabriz University of Medical Sciences, Tabriz, Iran.; ^2^Cardiovascular Research Center, Tabriz University of Medical Sciences, Tabriz, Iran.; ^3^Department of Food Science and Technology, Faculty of Nutrition, Tabriz University of Medical Science, Tabriz, Iran.; ^4^Research Center for Evidence Based Medicine (RCEBM), Tabriz University of Medical Sciences, Tabriz, Iran.; ^5^Physical Medicine and Rehabilitation Research Center, Aging Research Institute, Tabriz University of Medical Sciences, Tabriz, Iran.

**Keywords:** Traditional medicine, Hypertension management, Herbal medicine, Persian medicine, Cardiovascular diseases

## Abstract

Traditional medicine is a comprehensive term for ancient, culture-bound health care practices that existed before the use of science in health matters and has been used for centuries. Medicinal plants are used to treat patients with cardiovascular diseases, which may occur due to ailments of the heart and blood vessels and comprise heart attacks, cerebrovascular diseases, hypertension, and heart failure. Hypertension causes difficulty in the functioning of the heart and is involved in atherosclerosis, raising the risk of heart attack and stroke. Many drugs are available for managing these diseases, though common antihypertensive drugs are generally accompanied by many side effects. Medicinal herbs have several active substances with pharmacological and prophylactic properties that can be used in the treatment of hypertension. This review presents an overview of some medicinal plants that have been shown to have hypotensive or antihypertensive properties.

## Introduction

Cardiovascular diseases (CVDs) are a major cause of weakness and early death and, therefore, constitute a main communal health problem.^1^ High blood pressure (BP), mentioned as a silent killer, is triggered by a range of factors, including the interaction of genetic and environmental components causing disorderliness in BP regulation.^2^ Hypertension (HTN) is the most common risk factor in acute myocardial infarction and is accountable for about 16.5% deaths annually across the world. It is also the most important reason for the morbidity and mortality accompanying CVDs.^3^ It has been predicted that by the year 2025, 29% of the world’s adults, or almost 1.56 billion people, will suffer from HTN.^4^ HTN is described as systolic blood pressure (SBP) ≥ 140 mm Hg and diastolic blood pressure (DBP) ≥ 90 mm Hg, according to the mean of 2 or more appropriate measurements of seated BP.^5^ Many antihypertensive mediators are used for the treatment of HTN, such as diuretics, sympatholytic agents, renin inhibitors, angiotensin converting enzyme (ACE) inhibitors, calcium channel blockers, β-adrenergic and α_1_/β-adrenergic antagonists, and vasodilators.^6^ These drugs have various side effects, including muscle cramps, abnormal heart rate, blurred vision, skin rash, vomiting, kidney failure, extreme tiredness, headache, and edema.^7^ Current growth in the acceptance of alternative medicines and natural products has drawn attention to traditional medicines for the treatment of CVDs.^8^ Approximately 75% to 80% of the world’s population, predominantly in developing countries, uses herbal medicines for primary healthcare because of their better compatibility with the human body, lower costs than novel pharmaceuticals, and fewer side effects.^9^ Persian medicine, an ancient and well-known traditional system of medicine, is based on the theory of humors for the prevention and treatment of diseases.^10^ Persian medical scholars like Avicenna and Rhazes have described various types of diseases and recommended lifestyle modifications and herbal treatments for the alleviation of problems.

Medicinal plants have also been examined for their therapeutic properties. Some of them play an essential role in the production of over 50% of the currently available pharmaceutical drugs.^11^ In this review article, a review of the diverse plants that have antihypertensive effects for use in the management of HTN is presented.

## Pathophysiology of hypertension

The pathophysiological mechanisms implicated in the progress of HTN comprise raised vascular resistance, mainly distinguished through decreased vascular diameter because of enhanced vascular contraction and arterial remodeling.^12^ Numerous factors contribute to the pathophysiology of HTN, including increases in the renin-angiotensin-aldosterone system (RAAS), stimulation of the sympathetic nervous system, vasopressin, disturbed G protein-coupled receptor signaling, inflammation, different T-cell roles, and the diversity of vasoactive peptides secreted by other endothelial cells and smooth muscle cells.^13^ Increased arterial reactivity because of dysregulation in pro-oxidant enzymes and endothelial nitric oxide synthase (eNOS), increased basal and activated calcium levels via calcium channels, and co-occurrence of vascular smooth muscle cell (VSMC) hyperplasia and hypertrophy can cause enhanced vasoconstriction.^14^ Augmented vascular stiffness is conducive to HTN, and its problems, such as atherosclerosis, indicating that therapy must be focused on vascular stiffness instead of only the modulation of peripheral vascular resistance.^15,16^ Angiotensin II (Ang II) is able to stimulate cell cycle progress.^17^ Genetic diseases of renal sodium secretion, genetically associated ailments of the Na/Ca^2+^ exchange in the smooth muscles of arteries, and hormonal-neurogenic vasoconstriction are other possible causes of HTN. ^18^

## Herbal medicines used for the treatment of hypertension

**Figure 1:**
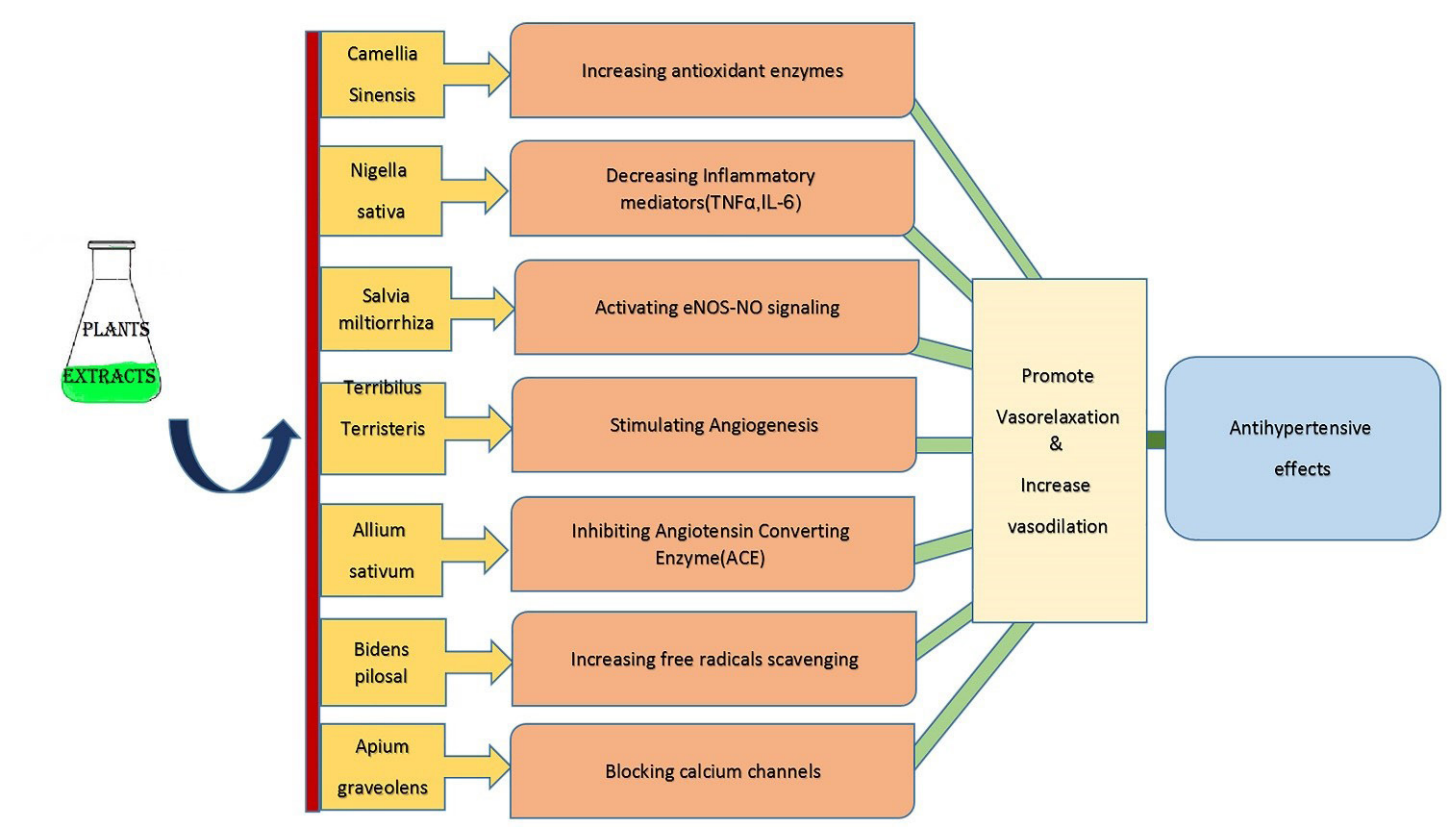
Schematic diagram showing the mechanisms of some of medical plants or their extracts in the management of hypertension.

Many antihypertensive agents usedin the treatment of HTN have some side effects. Therefore, scientific studies recommend diverse lifestyle alterations and the use of suitable medicinal plants in its treatment.^19^ Secondary metabolites of some herbs and spices display antihypertensive properties. Most herbal medicines control and reduce HTN by exerting antioxidant, anti-inflammatory, and anti-apoptosis properties, stimulating the eNOS-NO signaling pathway, suppressing endothelial permeability, and activating angiogenesis.^20^ The mechanisms of some medicinal plants or their extracts in the management of HTN are shown in Figure 1.

## Ajwain (Carum copticum L.)

*Carum copticum* belongs to the Apiaceae family and grows in various regions of Central Europe, Iran (particularly the eastern areas of Baluchistan), India, Afghanistan, and Pakistan.^21^ As a result of its calcium channel blocking effect, *C. copticum* has a notable role in regulating heart rate and BP. The aqueous-methanolic extract of *C. copticum*Benth. seeds (CSE) (1-30 mg/kg) causes a decrease in BP and heart rate (HR) of normotensive (NMT) rats. At larger doses (10-30 mg/kg), bradycardia has been reported.^21^

## Bindii (Tribulus terrestris)

*Tribulus terrestris* is a medicinal plant used for treating HTN. Bindii causes a decrease in BP in spontaneously hypertensive (SHR) rats. Its methanolic and aqueous extracts (0.3–15 mg/mL) have been shown to have vasodilatory properties.^22^ This plant is used for its diuretic effects. Furthermore, all of the saponins (furostanol and spirostanol saponins and sulphated saponins of tigogenin and diosgenin) of this plant prevent the production of H_2_O_2_ along with the proliferation of VSMCs.^23^

## Black Cumin (Nigella sativa)

The Nigella sativa plant, well recognized as the seed of blessing, has been used in the Middle East, Europe, and Africa for years. This plantand its components cause a decrease in BP.^24^ Oral administration of * N. sativa* seed oil extract (100 or 200 mg) to mild hypertensive male patients for eight weeks results in a decline of 10.6 and 9.6 mm Hg in SBP and DBP, respectively.^25^ Black cumin also lowers BP through vasorelaxation by means of its ability to block Ca^2+^ channels. Other mechanisms that may elucidate the hypotensive effect of *N. sativa* relate to its diuretic function, antioxidant activities, and anti-inflammatory properties.^26^

## Black-Jack (Bidens pilosa L.)

Black Jack, from the Asteraceae family, is an annual plant that grows in South America and is also found in tropical and subtropical regions around the world. Black Jack leaf extract was able to inhibit and reduce HTN in different rat models.^27^ In fructose-fed rats, six hours after treatment with 75 and 150 mg/kg of methanolic leaf extract, SBP was decreased by 17% and 21%, respectively.^27^ Additionally, *B. pilosa*has anti-cancer and anti-obesity effects as well as radical scavenging ability.^27^

## Black plum (Vitex doniana)

After oral administration of the fresh black plum fruit, both SBP and DBP were considerablydiminished in 45 minutes.BP began returning to standard after 2 hours.^28^

## Greater burdock (Arctium Lappa)

Burdock is also used for the treatment of HTN. This plant has reactive oxygen species (ROS) scavenging action, is able to inhibit vascular inflammation, and can stimulate vasorelaxation.^29^ Arctigenin (a dietary phytoestrogen) is one bioactive component in the dry seeds of burdock that causes an increase in NO production and a decrease in the levels of superoxide anion.^30^

## Burhead (Echinodorus grandiflorus)

*Echinodorusgrandiflorus* is used in Brazilian folk medicine as a diuretic drug. The aqueous extracts of this plant can cause a decline in the mean arterial pressure (MAP) in addition to cardiac output and vascular resistance in SHRs. Burhead also induces persistent diuresis and decreased BP by activating muscarinic and bradykinin receptors with effects on prostaglandins and nitric oxide pathways.^31^

## Cardamom (Elettaria cardamomum)

*Elettaria cardamomum* fruit powder has been assessed for its antihypertensive capability. In powder form (3 g), it has been shown to reduce mean MAP as well as SBP and DBP by 19 and 12 mm Hg, respectively in pre-hypertensive subjects by increasing the total antioxidant status.^32^

## Carrot (Daucus carota L.)

Carrot has been used in traditional medicine as an antihypertensive mediator. *Daucus carota L.* improves endothelial function and regulates fluid balance. Carrot juice is rich in antioxidants, which decrease oxidative stress and control the function and structure of blood vessels. Carrots regulate BP because of the existence of potassium. Intravenous administration of the bioactive components of the aerial parts of *D. carota,* including DC-2 and DC-3, triggered a decrease in arterial BP in NMT rats. DC-2 and DC-3 can act by obstructing calcium channels.^12^

## Cat’s Claw herb (Uncaria rhynchophylla)

Cat’s claw is an herb used in traditional Chinese medicine to treat HTN. This plant causes a decrease in BP and relieves different neurological symptoms. Hirsutine (an indole alkaloid) is responsible for thehypotensive function of *Uncariarhynchophylla*, which decreasesintracellular Ca^2+^ levels through its effect on the Ca^2+^ store and its effects on the voltage-dependent Ca^2+^ channel.^33^

## Celery (Apium graveolens)

The seed extract of celery has been shown to have a BP-reducing effect in deoxycorticosterone acetate (DOCA)–induced hypertensive rats. The hexane extract is considerably more effective in reducing BP, probably by reducing levels of circulating catecholamines and diminishing vascular resistance. Extraordinarily, it has antioxidant effects due to the virtue of its flavonoid content.^34^

## Chakshushya (Cassia absus L.)

*Cassia absus* is a plant of the family Fabaceae with Ayurvedic ethnomedical records. This plant occurs in tropical areas and all over India. Intravenous administration of the alkaloid isolated from the seeds of Cassia absus Linn (1-30 mg/kg) reduces BP in rats. At higher doses (10 and 30 mg/kg), it causes a decline in HR. Frequent injection of a similar dose induces tachyphylaxis.^35^

## Chinese Sage (Salvia miltiorrhiza)

A traditional Chinese herb, *Salvia miltiorrhiza*, has been revealed to have cardioprotective effects on animals and humans. In addition to its vasodilatory capability, Chinese sage possesses anti-hypertensive properties including antioxidative effects through decreased ROS production, increased antioxidative enzymes, and anti-proliferative activities by preventing platelet-derived growth factor (PDGF)-induced proliferation of VSMCs, and anti-inflammatory capacity by inhibiting TNF-α and NF-κB production.^36,37^

## Cinnamon (Cinnamomum zeylanicum)

Another plant used for the treatment of HTN is *Cinnamomum zeylanicum*. Cinnamon has reduced BP in numerous rat models and in people with prediabetes and type2 diabetes (T2D). The aqueous extract of its stem bark causes a reduction in SBP and prevents contractions prompted by potassium chloride (also known as KCl), related to the endothelium, NO, and ATP-sensitive K+ channel (K ATP channel). The methanolic extract of the bark increases NO levels.^38^

## Cocoa Bean (Theobroma cacao)

Cocoa powder, augmented with flavonoidcomponents, is used for inhibiting CVDs by motivating the creation ofNO, increasing vasodilatation, and decreasing endothelialdysfunction. Daily use of dark or milk chocolate (40 to 105 g) can decreaseSBP by about 5 mm Hg and DBP by about 3 mm Hg.^39^

## Coffee Weed (Cassia occidentalis)

Coffee weed also decreases BP. The leaf of this plant is used as an antihypertensive agent. Coffee weed has been found to decrease BP levels, probably through the suppression of external Ca^2+^ influx. Coffee weed leaves have diuretic effects along with anti-inflammatory and anti-oxidant properties. They decrease lipid peroxide content and inhibit phospholipase A2 activity.^40^

## Coriander (Coriandrum sativum)

Coriander is used as a traditional medicine for the treatment of cardiovascular and gastrointestinal diseases. It has been shown to display antioxidant effects.^41^ Intravenous use of the aqueous methanolic extract of the seeds (1–30 mg/mL) causes a reduction in SBP, DBP, and MABP, possibly through the Ca^2+^ antagonist. Additionally, this extract exhibits diuretic affects.^42^

## Dogbane (Apocynum venetum)

The leaves of the dogbane plant seem to be rich in flavonoids and quercetin variants, which have been found to help fight HTN. Extracts of dogbane leaves (10 μg/mL) induce vasorelaxation by enhancing NO, causing the scavenging of ROS. This plant’s extracts improve renal function as an antihypertensive effect.^43^

## Dog-strangling Vine (Cynanchum wilfordii)

*Cynanchum wilfordii* is used in traditional Chinese medicine, and nearly all parts of this plant are considered advantageous for different vascular diseases. Ethanolic extracts (100 and 200 mg/kg/d) of *C. wilfordii* reduced BP in high fat/cholesterol-fed rats, by motivating Akt, triggering increased eNOS activity as well as increased NO and cyclic guanosine monophosphate (cGMP) production in addition to a decline in the expression of VCAM-1 and endothelin-1 (ET-1).^44^

## Harmel (Peganum harmala)

Wild Syrian rue (family Zygophyllaceae) is called “Espand” in Persian, and different parts of this plant including its seeds, bark, and root have been used in folk medicine.^45^ Espand is used for the treatment of HTN. *Peganum harmala* prompts relaxation through both endothelial cells and VSMCs. Three harmala alkaloids, i.e. Harmine, harmaline, and harmalol, are espand’s active constituents which have shown vasodilatory properties by increasing NO production.^46^

## Fang Ji (Stephania tetrandra)

*Stephania tetrandra* is able to regulate high BP by reducing inducible nitric oxide synthase (iNOS) expression and blocking Ca^2+^ channels. An alkaloid tetrandrine, the bioactive constituent of this plant, has anti-inflammatory and anti-oxidant effects, both of which are probably involved in the plant’s antihypertensive effects.^47^

## Garden Cress (Lepidium sativum L.)

The hypotensive effect of garden cressis associated with the augmented urinary removal of sodium, potassium, and chlorides. *Lepidium sativum* has been revealed to have anti-inflammatory effects. *Lepidium sativum* induces diuresis and effective antioxidant capability, to which its antihypertensive effects may be ascribed.^48^

## Garden Nasturtium (Tropaeolum majus L.)

Garden nasturtium belongs to the family Tropaeolaceae. Studies have confirmed that *Tropaeolum majus* has a positive influence on the circulatory system. Hydroethanolic extracts of garden nasturtiumhave been revealed to decrease MAP in SHR rats. The ethanolic extract of *T. majus*(300 mg/kg), cure element (100 mg/kg), or isoquercitrin (10 mg/kg), have diuretic activities. All the above-mentioned constituents are able to reduce plasma ACE levels. Isoquercitrin (an active flavonoid) causes the growth of NO production.^49^

## Garlic (Allium sativum)

Garlic supplements have revealed their effectiveness in the treatment of HTN, decreasing BP by about 10 mm Hg systolic and 8 mm Hg diastolic, like standard BP medication. This herb is recognized for its antibacterial, antioxidant, anti-inflammatory, anti-cancer, and hypocholesteremic effects.^50^ One study displayed that garlic had an approximately 80% effectiveness in the treatment of HTN. Aged garlic extract (AGE) induces a constant drop in BP compared to with other forms of garlic. Furthermore, garlic supplements prompt a major decrease in both SBP and DBP by 3.75 and 3.39 mm Hg, respectively.^51^ In another study, patients with HTN who ingested garlic tablets (300–1500 mg/d) for 24 weeks described a considerable reduction in SBP by 9.2 mm Hg and DBP by 6.27 mm Hg.^52^ Moreover, AGE has superoxide scavenging abilities in human neutrophils, and daily use of 150 or 400 mg/kg of garlic extract prompted an increase in eNOS activity and a decline in nicotinamide adenine dinucleotide phosphate (NADPH)-oxidase in the aortas of fructose-fed rats.^53^ The components of garlic inhibit ACE activity, diminish Ang II-induced vasoconstrictor responses, prevent VSMCs proliferation in smooth muscles, antagonize endothelin-1 prompted vasoconstriction, and inhibit the stimulation of NF-κB.^54^

## Giant dodder (Cuscuta reflexa)

Cuscuta, commonly known as dodder, is a genus of the family convolvolaceace. The ethanolicextract of *C. reflexa* Led to a decline in SBP and DBP in anesthetized rats. In a dose-dependent manner, antihypertensive activity and bradycardia occurred.^55^

## Ginger (Zingiber officinale)

*Zingiber Officinale*, generally recognized as ginger, has been broadly used in the daily diet and for different therapeutic purposes. Ginger contains a large amount of potassium, which plays a role in the regulation of BP and heartbeat. Administration of two bioactive components of ginger, (6)-gingerol and (6)-shogaol, orally (70– 140 mg/kg) or intravenously (1.75–3.5 mg/kg) creates tri-phasic BP profiles: first a rapid drop, then an intermediate increase, and lastly, a delayed decline in BP. Currently, (6)-gingerol is considered to be a new Ang II type 1 receptor antagonist.^56^ Recently, it has been found that ginger decreases levels of total cholesterol, triglycerides, low-density lipoprotein (LDL), and very low-density lipoproteins (VLDL). It also inhibits ACE-1 activity.^57^

## Ginseng (Panax spp.)

Ginseng is used in different forms, either as capsules, tablets, extracts, dried roots, oil, or as tea, and has hypotensive effects.^58^ Small doses of ginsengincrease BP, whereas higher doses are hypotensive. Thus, ginsengregulates BP levels in hypotensive patients probably through vascular function change, controlling the autonomic nervous system, or adjusting the arterial baroreflex.^58^ The * panax* ginsengextract in mild hypertensive patients induces a considerable decline of 3.1 mm Hg in SBP and mm Hg in 2.3 DBP.^59^ Ginsenoside Rg3(red ginseng) stimulates eNOS, enhances NO and cGMP levels, and stimulates Ca^2+^ - gated K^+^ channels. Moreover, ginsenghas an anti-proliferative influence on VSMCs, and it has antihypertensive and anti-atherosclerotic abilities.^60^ Red ginseng also reduces Ang II-induced VSMC growth. Another hypotensive mechanism of ginseng is its antioxidant ability, perhaps by increasing antioxidant enzymes and scavenging free radicals. Furthermore, ginsengdisplays anti-inflammatory properties by protecting the release of TNF-α and decreasing NF-κB and p38 MAPK pathways.^60^

## Goldthread (Coptis chinensis)

Goldthread and its most important constituent Berberine (BBR) can decrease BP. *Coptis chinensis* can block Ca^2+^ channels and inhibit cardiac hypertrophy. BBR can cause a significant decline in SBP (by an average of 4.91 mm Hg) and DBP (2 mm Hg).^61^ BBR (150 mg/kg) can also scavenge ROS, prevent NADPH oxidase, and increase the antioxidant enzymes and superoxide dismutase (SOD).^62^ BBR increases the expression of eNOS with a simultaneous increase in NO release that causes increased vasodilation. Furthermore, BBR prevents endothelial injury and controls inflammatory pathways by inhibiting NF-κB, VCAM-1expression and VSMC proliferation.^62^

## Gumbo limbo (Bursera simaruba)

*Bursera simaruba*, usually known as gumbo-limbo, is native to the tropical regions of the Americas. *B. simaruba* was shown to decrease heart rate and cause long-standing hypotension after one oral administration of the extract*. B. simaruba* also advanced the endothelial function by activating vascular endothelial NO synthase, thereby explaining the plant’s vascular protective influence.^63^

## Hardy fuchsia (Fuchsia magellanica)

*Fuchsia magellanica* is found in Chile and Southern Argentina. The leaf extract of this plant decreases body heat, has diuretic effects, and reduces BP. In NMT rats, ethanol/aqueous extracts of this species caused a significant decrease in MAP.^64^

## Hawthorn (Crataegus spp.)

Hawthorn plants have been used for the treatment of CVDs. Patients with mild HTN who were treated with 500 mg of hawthorn extract for ten weeks displayed a decrease by 13.1 mm Hg decrease in DBP.^65^ Quercetin, a main compound in hawthorn shrubs, has antioxidant, anti-inflammatory, and vasorelaxant effects. Remarkably, hawthorn extracts affect both VSMCs and endothelial cells.^66^ Furthermore, they have anti-inflammatory activity by decreasing the concentrations of NF-κB, TNF-α, VCAM-1, iNOS, and IL-6.^67^

## Indian Plantago (Plantago ovata)

Indian Plantago is an herb, the seed and outer covering of the seed (husk) of which are used to make medicine. A primary clinical study indicated that using 15 gof *Plantago ovata* supplement everyday could relatively reduce BP: SBP by around 8 mm Hg and DBP by 2 mm Hg.^68^

## Indian snakeroot (Rauwolfia serpentina)

*Rauwolfia serpentina* is a tropical woody plant used for the treatment of HTN by reducing the levels of dopamine and epinephrine and by promoting vasodilation. Reserpine, the main alkaloid of *Rauwolfia serpentina*, is the primary powerful drug broadly used in the longstanding treatment of HTN. In 1952, isolated reserpine was made known as the drug Serpasil for the treatment of HTN, tachycardia, and thyrotoxicosis.^69^

## Japanese Thistle (Cirsium japonicum)

*Cirsium japonicum* is a perennial herb native to Japan, China, and Korea. The aqueous extract (0.1–1.0 mg/mL) of this plant induces vasorelaxation by activating histamine H_1_ receptors. The principal mechanism includes elevated levels of NO and cGMP. Silibinin (0.05–0.4 mg/mL), a component of Japanese thistle, is an antagonist for the human angiotensin II receptor type 1 (AT_1_ receptor), so it can reduce SBP.^70^

## King of Bitters (Andrographis paniculata)

King of bitterhas been used in Asian traditional medicine for the treatment of CVDs.^71^ The extracts of *A. paniculata* has been shown to lower ACE and ROS activities in SHR rats and cause a reduction in BP. The crude extract of *A. paniculate*, a compound of 14-deoxy-11,12-didehydroandrographolide, prompts considerable hypotensive properties by increasing NO release and inhibiting the rise in intracellular Ca^2+^.^72^ It has been revealed to have anti-inflammatory, anti-bacterial, and antioxidant effects.^72^

## Kudzu (Pueraria lobata)

*Pueraria lobata* reduced BP in dogs and hypertensive patients through its vasodilatory effect, with its ability to stimulate Ca^2+^-activated K^+^ (KCa)channels.^73^ This plant has anti-inflammatory and anti-oxidant activities, which can relatively explain its anti-hypertensive effects. Puerarin is the major bioactive compound in this plant which has antihypertensive and other cardioprotective properties.^74^

## Large-fruited elm (Ulmus macrocarpa)

Oral administration of 100 mg/kg root bark of *Ulmus macrocarpa* (RBUM) reduced SBP in SHR rats by 20 mm Hg. The anti-hypertensive influence of RBUM could be due to its ability to improve structural and functional modifications of vascular endothelium.^75^

## Lemongrass (Cymbopogon citratus)

Lemongrass is a plant whose leaves and oil are used to make medicine. Lemongrass is widely used in Southern Asia, China, and Brazil. Its antihypertensive effects have been ascribed to Citral, its active phytochemical compound.^76^ Citralor crude extracts cause dose-dependent vasorelaxation through the activation of NO and the suppression of calcium channels. Lemongrass exerts modest antioxidant activity by suppressing ROS molecules and is involved in anti-inflammatory pathways by preventing NF-κB and iNOS activity.^77^

## Logolai (Bag.) (Viscum articulatum Burm.f.)

*Viscum articulatum* Brum.f. methanolic extract has diuretic properties. Furthermore, oleanolic acid extracted from *Viscum articulatum* Brum.f.(60 mg/kg/d) can raise NO levels in plasma. This plant also has antioxidant potential.^78^

## Makandi (Coleus forskohlii)

The* Coleus forskohlii* plant is a strong adenylyl cyclase activator. Makandi raised intracellular levels of cAMP, triggering the activation of protein kinase A(PKA), which consecutively prompted the relaxation of VSMCs, thereby causing a decrease in BP.^79^

## Maritime Pine (Pinus pinaster)

Pycnogenol (200 mg/d), an extract from French maritime pine bark, can relatively reduce BP inpeople with mild HTN, possibly by preventingangiotensin-converting enzymes.^80^

## Mistletoe (Agelanthus dodoneifolius)

Ethanolic extracts of mistletoe (0.01–10 mg/mL) reduced SBP and DBP in normotensive rats. The dodoneine mechanism induced vasorelaxation by preventing carbonic anhydrase and stimulating KCa channels.^81^

## Melon-Gubat (Melothria maderaspatana)

*Melothria maderaspatana* causes a reduction in BP in hypertensive humans. The use of melon-gubat tea for 45 days in subjects with mild HTN resulted in a substantial reduction by 23.8 mm Hg and 15.5 mm Hg in systolic and diastolic BP, respectively.^82^

## Murungai (Moringa oleifera)

The crude extract of the leaves of the *Murungai* plant triggers a decreasein SBP, DBP, and MBP in a dose-dependent manner by lessening vascular dysfunction and oxidative stress and stimulating endothelium-dependent vasorelaxation.The antihypertensive activity has been attributed to the thiocarbamate andisothiocyanate elements of the purified extract.^83^

## Onion (Allium cepa)

Onion was shown to decrease BP in fructose-fed and anesthetized normotensive rats.^84^ Organo-sulfur compounds have been correlated with reducing BP by sustaining the elasticity of the major arteries accompanied by lowering the blood viscosity, thereby preventing blood clotting.^85^ Quercetin, the composite most usually related to onions, can decrease BP an average of 5 mm Hg by decreasing oxidative stress through its reaction with free radicals and progressing vascular function.^85^ Aqueous extracts of onion (400 mg/kg/d) increased eNOS expression but decreased that of VCAM-1. The antioxidant effects of onion seem to be the result of the inhibition of NADPH oxidase activity together with a simultaneous rise in antioxidant kinetics of glutathione peroxidase (GPX) enzymes and SOD.^86^

## Pointed Phoenix Tail (Gynura procumbens)

In Thai, *Gynura procumbens* is called “longevity spinach,” and in Chinese, it is called “Pointed Phoenix Tail.” The aqueous extract of pointed phoenix taildecreases BP in SHRs. In rat aortic rings, it inhibited contractions induced by Ang I and Ang II through a NO-dependent mechanism and inhibited ACE activity.^87^ Furthermore, the crude extract of this plant (0.003 and 0.009 g/mL) suppressed both KCl- and phenylephrine-induced contractions, associated with the opening of K channels, preventing the Ca^2+^ channels and discharge of prostacyclin, so it displayed a vasodilatory effect.^88^

## Pomegranate (Punica granatum)

The pomegranate is a fruit-bearing deciduous shrub in the family Lythraceae that grows in the region extending from Iran to northern India. Pomegranatedecreases the activity of ACE by nearly 36%. One study displayed a modest decrease inSBP after drinking 50 ml/day of its juice for ayear.^89^

## Prickly Custard apple (Annona muricata).

*Annona muricata* is a species of the custard apple tree family. Annonaceae, which has edible fruit. *A. muricata* is native to Central America and the Caribbean. The methanolic extract of the *A. muricata* leaf has been described to decrease a raised BP by reducing peripheral vascular resistance.^90^

## Qingxue Dan (Chunghyul-dan)

*Chunghyul-dan* is an herbal complex that has anti-hypertensive effects on stroke patients with stage 1 HTN. In stroke patients, after the administration of 1200 mg *Chunghvul-dan*, SBP and DBP were considerably reduced in comparison with baseline.^91^

## Radish (Raphanus sativus)

Radish is an edible root vegetable of the family Brassicaceae, grown and used throughout the world. The leaf ethyl acetate extract (30 and 90 mg/kg) reduced SBP in SHRs, whereas the seeds (0.1–3 mg/kg) reduced BP along with HR. Radish extracts increase NO production and raise antioxidant levels. The anti-proliferative and anti-inflammatory capabilities of the radish may be partially involved in its antihypertensive effect.^92^

## Roselle (Hibiscus sabdariffa)

*Hibiscus sabdariffa* L. (HS) tea is used as a beverage and a treatment for HTN and hyperlipidemia. In patients with HTN, treatment with the dried extract of the calyx (250 mg) for 4 weeks has displayed remarkable antihypertensive effects.^93^ After four weeks of ingesting 10 g/d of hibiscus calyx, the SBP and DBP of hypertensive patients was decreased significantly by 15.32 and 11.29 mm Hg, respectively. In mild and pre-hypertensive patients, using hibiscus tea (240 ml) 3 times daily for six weeks decreased SBP, DBP, and MAP considerably by 7.2, 3.1, and 4.5 mm Hg, respectively.^94^ Its effects were facilitated through the elevated production of NO, blocking of Ca^2+^ channels, and opening of KATP channels. Roselle has diuretic effects, exhibits potent antioxidant function, and prevents the oxidation of LDL. Moreover, it shows anti-inflammatory capabilities through the prevention of ACE activity and proliferation of VSMCs.^95^

## Safflower (Carthamus tinctorius L.)

*Carthamus tinctorius* L., known as Kafesheh (Persian), is used extensively for numerous medical conditions including cerebrovascular and CVDs in traditional Chinese medicine. Safflower yellow (SY) reduced BP by opening KATP channels in addition to decreasing renin activity and Ang II levels in SHRs. In addition to reducing BP in healthy humans, the seed extract (2.1 g daily) also reduces both VCAM-1 and LDL levels, prevents PDGF-induced proliferation of VSMCs, and decreases arterial stiffness.^96^

## Saffron (Crocus sativus)

*Crocus sativus* L., generally known as saffron, is a fragrant plant belonging to the Iridaceae family. This plant is native to Spain, Morocco, Greece, Iran, India, and Pakistan.^97^ Administration of 400 mg of saffron tablets to healthy humans for seven days led to a decrease of 11 and 5 mm Hg in SBP and MAP, respectively. In male Wistar rats, crocin treatment (200 mg/kg for 7 days) caused a major drop in oxidative stress and an increase in antioxidant enzymes.^98^ Additionally, saffron and its components blocked the inflammatory pathways comprising NF-κB and TNF-α.^99^

## Sesame (Sesamum indicum)

Sesame is a flowering plant in the genus Sesamum. Sesame oil is a suitable prophylactic treatment for HTN. The alcoholic extract of the seeds (1–30 mg/kg) was shown to trigger a reduction in BP in anesthetized rats.^100^

## Shell Ginger (Alpinia zerumbet)

Shell Ginger, also known as bright ginger, is a perennial species of ginger from the family of Zingiberaceae.* Alpinia zerumbet*, a west Asian plant, has modest hypotensive properties. The vasorelaxant responses of methanolic fraction of the essential oil of shell ginger are induced through its effects on endothelial cells or VSMCs.^101^ In DOCA-salt-treated rats, the methanolic extract of this plant’s leaves (100 and 300 μg/mL) prompted vasodilation by raising NO or cGMP production. 1–20 mg/kg of *Alpinia zerumbet* essential oil blocks Ca^2+^ channels, and 0.1 mg/L of this oil causes a decrease in the levels of oxidized LDL in plasma.^102^

## Stone breaker (Phyllanthus niruri)

*Phyllanthus niruri* is a plant that causes a reduction in BP in rabbits and humans. The aqueous extract of stone breaker(200 mg/kg) raises plasma antioxidants (GSH, GPx, SOD, and catalase CAT). Additionally, diverse solvent extracts of stone breakerhave been reported to prevent NF-κB, TNF-α, and COX-2.^103^

## Sumac (Rhus coriaria)

Sumac is a medicinal plant traditionally used for the treatment of CVDs.* Rhus coriaria* is known for its antioxidant activity. Hydrolysable tannins obtained from the leaves of sumac have been reported to display a vasorelaxant effect in an endothelium-dependent and NO-mediated manner. Importantly, this extract also has effective anti-inflammatory capabilities and can cause a decrease in TNF-α.^104^

## Sweet basil (Ocimum basilicum)

*Ocimum basilicum* L. is an herb used in traditional Chinese medicine to treat CVDs. In one study, the aqueous extract reduced BP levels in rats in a dose-dependent manner (100–400 mg/kg). It also induced a vasorelaxant effect and had ROS scavenging ability.^105^

## Sweet flag (Acorus calamus)

Sweet flag is a commonly known drug in the traditional system of medicine. The solvent extracts of sweet flagcaused a reduction in MAP in normotensive rats. *Acorus calamus* has vasoconstrictive or vasodilatory properties in rabbit aorta as well, possibly due to a Ca^2+^dependent mechanism.^106^

## Sweet violet (Viola odorata)

*Viola odorata*, commonly known as sweet violet, is native to Europe and Asia. The leaf extract of sweet violet (0.1, 0.3, and 1 mg/kg) lowered the MAP of rats. The extract induces relaxation and NO production, and Ca^2+^ influx control causes this vasodilatory effect. The extract also improves CVDs risk factors by stimulating a substantial decline in total cholesterol and LDL-C.^107^

## Tea (Camellia sinensis)

Tea is a beverage of cured leaves or leaf buds of the tea plant *Camellia sinensis.*^108^ It has pleiotropic effects comprising antibacterial, anti-inflammatory, anti-cancer, and anti-diabetic properties, accompanied by antihypertensive actions. Green tea decreases both SBP and DBP by 1.98 and 1.92, respectively.^109^ Remarkably, it has been stated that green tea induces a more potent hypotensive effect than black tea.^110^ One study established that hypertensive patients who used up to 4479 mg of black tea for 24 weeks showed a substantial decrease by 2 and 2.1 mm Hg in SBP and DBP, respectively.^111^ The bioactive constituents of tea have been shown to exert anti-oxidant and anti-inflammatory effects. The mechanisms of oxidative stress reduction by tea which include increasing CAT antioxidant enzyme, inhibition of eNOS uncoupling, superoxides scavenging capacity, and reducing NAPDH oxidase production, cause a reduction in BP besides TNF-α levels.^108^ Epigallocatechin gallate (EGCG), derived from tea, caused a decrease in VCAM-1 levels, prevented NF- κB activation, and stimulated prevention of proliferation in human aortic VSMCs through the upregulation of HO-1 enzyme expression.^112^

## Tianma (Gastrodia elata Blume)

*Gastrodia elata* is a saprophytic perennial herb of the family Orchidaceae and is used in traditional Chinese medicine.*Gastrodia* rhizome has antihypertensive properties. The acidic polysaccharides extracted from the rhizome trigger a significant reduction in BP levels.^113^ The methanolic extracts (0.02 ml/g) of Tianmaexhibited anti-inflammatory properties by decreasing iNOS expression and NO levels. In old patients with refractory HTN, gastrodin, a main bioactive constituent of Tianma, triggered a decline in SBP and pulse pressures, raised NO levels, and decreased endothelin levels. Gastrodin (a phenolic glycoside) decreased SBP by interfering with the RAAS and diminished serum levels of Ang II along with the expression of both ACE and AT1R.^114^

## Tomato (Lycopersicon esculentum)

The tomato is the edible part of the plant Solanum lycopersicum. Tomato extract contains carotenoids which are recognized as operative antioxidants. The extract of tomato (Lyc-O-Mato) moderately decreased BP in patients with HTN.^115^ Tomato extract has a clinically substantial capacity to decrease SBP by more than 10 mm Hg and DBP by more than 5 mm Hg.^115^ The root extract of tomato reduced BP levels in hypertensive rats. The antioxidant-rich extract of tomato has been revealed to decrease both SBP and DBP in hypertensive patients.^116^

## Turmeric (Curcuma longa)

*Curcuma longa* or turmeric, originates from Southeast India and is widely cultivated in the tropical areas of South Asia. Turmeric, also called curcumin, has anti-inflammatory and anti-cancer properties.^117^ Curcumin exerts advantageous effects on CVDs, such as HTN. Curcumin decreases AT_1_R expression in arteries by disturbing SP1/AT_1_R DNA binding, thereby decreasing AT_1_R-mediated vasoconstriction and then inhibiting the progress of HTN.^118^

## Umbrella tree (Musanga cecropioides)

*Musanga cecropioides*, the African corkwood or umbrella tree, is found throughout the tropical rain forests, mostly in West Africa. The latex and the leaf extract of this plant are used as a vasorelaxant and a hypotensive mediator. The water extract of the stem bark produces a dose-dependent decrease in MABP at the dose of 10 mg/kg (4.51 ± 0.5 mm Hg) and at the 40 mg/kg dose (65.23 ± 6.28 mm Hg).^119^

## Vidanga (Embelia ribes)

*Embelia ribes*, commonly known as false black pepper, is a species in the family Primulaceae. It is widely dispersed throughout India.*Embeliaribes* has hypotensive effects. The aqueous extract of *E. ribes* (100 mg/kg) is able to reduce both SBP and HR and enhance endogenous antioxidants, including SOD, CAT, and GSH.^120^

## White Horehound (Marrubium vulgare)

*Marrubium vulgare* (common horehound) is a flowering plant native to Europe, northern Africa, and southwestern and central Asia. White horehound causes a significant decrease in SBP. This hypotensive effect may be due to its anti-hypertrophic and vasorelaxant properties. The diterpene marrubenol isolated from this plant can strongly block L-type Ca^2+^ channels and subsequently prevent the contraction of VSMCs. Also, phenylpropanoids extracted from white horehound can prevent the lipoprotein-induced secretion of endothelin-1.^121^

Some medicinal plants which are commonly acknowledged to be beneficial in the treatment of HTN are discussed in Table 1.

**Table 1:** Effective medicinal plants on hypertension

**Herb**	**Mechanism of Action**	**Part used**	**Dose**	**References**
Ajwain *(Carum copticum)*	-Blocks calcium channel - Cholinomimetic effects - Causes to vasodilation of coronary arteries -Decreases systemic blood pressure	-Leaves -Seed-like fruit	1-30 mg/kg	^21^
Bindii (*Tribulus terrestris*)	- Increases NO - Reduces ACE - Inhibits Ang II-induced proliferation	-Leaves -Aqueous extract of tribulus fruits	0.3–15 mg/mL	^23^
Black Cumin (*Nigella sativa*)	-Reduces in cardiac oxidative stress -Reduces angiotensin-converting enzyme activity -Increases in cardiac heme oxygenase-1 activity -Prevents of plasma nitric oxide loss	-Seeds oil	100 mg/kg and 200 mg/kg	^25^
Black-Jack(*Bidens pilosaL*)	-Is calcium channel antagonism	-Leaves	75 and 150 mg/kg	^27^
Burdock(*Arctium Lappa*)	- Suppresses VCAM-1 (aortic endothelia) - Promotes vasorelaxation	- Root	100 and 200 mg/kg/d	^29^
Cardamom(*Elettaria cardamomum*)	- Blocks Ca^2+^ channels - Increases urine output -Enhances Na+ and K+ excretion	- Crude	3 g/d	^32^
Celery(*Apium graveolens*)	-Decreases levels of circulating catecholamines -Reduces vascular resistance -Blocks calcium channel	-Seeds	300mg/kg	^34^
Chinese Sage(*Salviae miltiorrhizae*)	- Increases NO - Opens K_ATP_ channels - Blocks Ca^2+^ channels - Reduces ACE activity	- Dried root	0–10 mg/mL	^37^
CocoaBean(*Theobroma cacao*)	-Up-regulates NO -Promotes vasodilation -Improves endothelial function	- Cocoa Bean	40 - 105 g	^39^
Garden Nasturtium(*Tropaeolum majus L*)	- Downregulates ACE - Increases NO - Decreases aldosterone - Reduces renal Na+/K+ pump - Enhances urine volume	-Seeds -Leaves -Flowers	10-300 mg/kg	^49^
Garlic(*Allium sativum*)	-Relaxes of blood vessels -Reduces in the ability of blood to clot - Increases NO - Inhibits ACE - Prevents Ang-II-induced cell cycle progression	-Fruits	300–1500 mg/d	^52^
Ginger(*Zingiber officinale*)	-Blocks Ca^2+^ channels - Promotes vasodilation	- Root	70– 140 mg/kg	^56^
Ginseng **(***genus Panax***)**	-Enhances NO and cGMP levels -Has an anti-proliferative influence on VSMCs	- Root	3 g/d	^60^
Japanese Thistle(*Cirsium japonicum*)	-Induces vasorelaxation -Elevates levels of NO -Is an antagonist for the AT1 receptor	-Whole plant	0.05–0.4 mg/mL	^70^
Onion* (Allium cepa)*	-Elasticity of arteries -Decreases in blood viscosity -Interfaces with Renin-Angiotensin System -Improves of endothelial and vascular function	-Fruits	400 mg/kg/d	^85^
Pomegranate *(Punica granatum)*	-Enhances endothelium-dependent coronary relaxation -Inhibits of calcium influx -Reduces ACE activity	-Fruits	50 mL/d	^89^
Radish(*Raphanus sativus*)	- Increases NO production	-Seeds -Leaves -Root	30 and 90 mg/kg	^92^
Roselle(*Hibiscus sabdariffa*)	- Enhances production of NO -Inhibits of Ca2+ channels -Opens of K_ATP_ channels	-Leaves -Flowers	250 mg-10 g/d	^94^
Saffron(*Crocus sativus*)	- Activates eNOS - Blocks Ca^2+^ channels	-Stigma	400 mg	^98^
Sumac(*Rhus coriaria*)	- Evokes endothelium-dependent vasorelaxation - Activates eNOS	- Leaves - Fruits (red berries)	0.3–300 μg/mL	^104^
Tea(*Camellia sinensis*)	-Inhibition of angiotensin converting enzyme - Blocks Ca^2+^ channels - Diuretic -Enhances eNOS activity	- Leaves	3 cups/day	^108^
Turmeric (*Curcuma longa*)	-Interference with Ca^2+^ concentration - Reduces ACE activity - Reduces AT1 receptor expression -Increase vasodilation -Increase No production	- Root	50-100 mg/kg/d	^118^

## Medicinal plants used for the treatment of HTN in Iran

The Sassanid Empire in Iran had an efficient and advanced official system of medicine that prominently influenced the advancement of medical sciences.^122^ For the duration of the golden age of the Islamic era, from the 9th to the 12th centuries A.D., medical information from numerous fields concerning cardiology thrived because of outstanding Persian physicians and scholars.^123^ Avicenna assumed and demonstrated that some natural medicaments have the capacity to help other treatments by directing them towards specific body organs.^124,125^ In view of that, Avicenna suggested the combination therapy of a cardiac medicine with Lemon balm (*Melissa officinalis* L.) or Behmen (*Centaurea behen* L.).^122^ The com­parison of herbal medicines used in studying HTN in different regions of Iran has indicated that different parts of Iran use diverse plants to treat this disorder.^126^ In Mobarakeh of Isfahan,curly dock *(Rumex crispus* L.*),*jujube (*Ziziphus jujuba* L.*),* and olive *(Olea europaea* L.*)* are used conventionally.^127^ In Sistan and Baluchestan prov­ince, nigella (*Nigella sativa* L.) is used to treat HTN.^128^ Milk thistle (*Silybum marianum* L.*),*yarrow* (Achillea tenuifolia),* chicory* (Cichorium intybus),* barberry* (Berberis vulgaris),* shepherd’s purse* (Capsella bursa-pastoris),* field horsetail* (Equisetum arvense),* Persian walnut* (Juglans regia),*and annual yellow sweetclover *(Melilotus indicus)*are used in Kazerun to treat HTN.^129^ In the Arasbaran region, barberry,yarrow* (Achillea millefolium* L*.),* ecballium* (Ecballium elaterium),* common hawthorn* (Cra­taegus monogyna)*, and English yew *(Taxus baccata* L*.)* are considered to have BP lowering potential.^130^ Falcaria vulgaris (*Falcaria vulgaris),*saffron* (Crocus haussknechtii),* berberidaceae* (Ber­beris integerrima),* Christ’s thorn jujube, ramsons* (Allium ursinum),* salsify* (Tragopogon porrifolius),* and dill *(Anethum graveolens)* are used to treat high BP in Lorestan Province.^131^ Warty-leaved rhubarb *(Rheum ribes* L*.)*and Christ’s thorn (*Paliurus spina-christi)* are used to decrease BP in Ilam province.^132^ The bioactive substances of dill could be a source for anti-HTN and anti-diabetes properties.^133^ Barberry has a lowering effect on BP. Valerian has BP diminishing effects in animals.^134^ Medicinal plants that have been recognized as being effective in controlling and treating HTN are listed in Table 2.^128,134,135^

**Table 2:** Complete information of therapeutic effects of medicinal herbs in high BP in Iran

**Scientific name**	**Persian Name**	**Usable Part**	**Region/Province**	**Preparation methods**
*Achillea millefolium* L.	Boumadaran	Shoot	East Azerbaijan (Arasbaran)	Decoction
*Allium sativum* L.	Sir	Root/ Bulb	West Azerbaijan	Fresh
*Allium ursinum*	Valak	Shoot	Lorestan	Raw or with food
*Althea aucheri* Boiss.	Khatmi-Armanestani	Aerial parts	Fars	Decoction
*Anthemis cotula* L.	Babouneye bahari	Flower	North of Iran	Decoction
*Anethum graveolens dhi*	Shevid	Whole parts	Lorestan	Dried or fresh with food
*Amygdalus scoparia*	Badam	Fruit	Lorestan	Sodden peel
*Berberis vulgaris* L. **/***Berberis integrima*	Zereshk	Leaves and fruit	East Azerbaijan (Arasbaran) / Lorestan	Cooked or sodden
*Camellia sinensis*	Chay-sabz	Leave	North of Iran	Decoction
*Capparis spinosa*	Hendevaneh aboujahl	Leaves and fruit	Lorestan	Raw fruit or dry leaf distillate is eaten
*Centaurea depressa* M.	Golegandom	Seed	Fars	Decoction
*Cichorium intybus* L.	Kasni	Leave	East Azerbaijan	Decoction
*Coriandrum sativum*	Geshniz	Leave	East Azerbaijan	Fresh
*Cotoneaster persica* Pojark.	Shirkhest	Aerial parts	Fars (Kuh-Delu)	Decoction
*Crataegus monogyna* /* Crataegus pontica* C. Koch.	Zalzalak	Leaves and fruit	East Azerbaijan (Arasbaran) / Ilam	Fresh or cooked or sodden
*Descurainia sophia* (L.) *Schr.*	Khakshir	Fruit	East Azerbaijan	Fresh
*Echium amoenum* L.	Gav zaban	Flower	Isfahan (Mobarakeh)	Decoction
*Falcaria vulgaris*	Ghaziaghi	Flower leaf, stem	Lorestan	Leaves are cooked and eaten with food
*Ficus religiosa*	Anjir	Fruit	Fars/ Lorestan	Fresh
*Glaucium oxylobum*	Shaghayegh-Goltiz	Leave	Golestan/Khorasan	Decoction
*Glaucium grandiflorum*	Shaghayegh-Goldorosht	Leave	Golestan/Khorasan	Decoction
*Gundelia tournefortii* L.	Kangar	Leave	Kurdestan	Fresh
*Hypericum perforatum*	Chay-Koohi	Leave	Kurdestan/ Azerbaijan / Ilam	Decoction
*Juglans regia* L.	Gerdou	Leaves and fruit	West Azerbaijan	Fresh
*Morus alba*	Toot	Fruit	Lorestan	Raw berry or dried berry
*Matricaria recutita*	Babooneh	Flower	Fars	Decoction
*Nasturtium officinale* R.	Alafe cheshme	Shoot	Kurdestan(Marivan)	Decoction
*Nectaroscordeum tripedale/ Nectaroscordeum coelzi*	Piaze tabestaneh lorestani	Shoot	Lorestan	Fresh
*Nigella sativa* L.	Siah daneh	Seed	Sistan	Fresh
*Olea europaea* L.	Zeytoon	Leave and Fruit	North Iran	Decoction
*Paliurus spina-christi* Miller.	Siah tale	Fruit	Ilam	Fresh
*Petroselinum sativum*	Jafari	Leave	East Azerbaijan	Fresh
*Physalis alkekengi*	Aroosak-Poshtpardeh	Aerial parts	Khuzestan	Decoction
*Rheum ribes* L.	Rivas	Stem	Ilam	Fresh
*Rhus Coriaria.* L.	Somagh	Fruit	Kurdestan	Decoction
*Ribes divaricatum /Ribes orientale*	Angoor	Leave and Fruit	East Azerbaijan (Maragheh/Arasbaran)	Fresh
*Rumex pulcher* L.* / Rumex crispus* L.*/ Rumex conglomerates* Murr	Torshak	Root/ Leaves and stem	Khuzestan/ Isfahan	Fresh
*Securigera securidaca* Degen & Dorfl.	Adas talkh	Seed	Khuzestan	Fresh
*Silybum marianum* L.* / Silybum marianum* (L.)Gaerth.	Khar maryam	Stem and root/ Flower	Khuzestan/Fars	Decoction
*Smyrnium cordifolium*	Andol	Seed	Lorestan	Squeezed seeds
*Suaeda altissima*	A type of Siah shor	Leaves and stem	North East Persian Gulf	Decoction
*Tragopogon aureus* Boiss	A type of Sheng	Leaves and fruit	Khuzestan	Fresh
*Trigonella monspeliaca*	Shanbalileh-Monileei	Leave and fruit	Fars	Fresh
*Valeriana officinalis*	Sonboletib	Aerial parts	Isfahan	Decoction
*Viscum album*	Darvash	Aerial parts	Khuzestan	Decoction
*Ziziphus jujuba* (L)H.Karst	Anab	Fruit	Isfahan	Fresh
*Ziziphus nummularia*	Konar	Bulb	Lorestan	Fresh
*Ziziphus spina-christi*	Sedr	Leaves, flowers and fruit	Lorestan	Sodden leaves and flower

## Study limitations

A small number of traditionally used plants have been confirmed precisely through animal studies and clinical trials, but the detailed mechanisms of action of these plants are still unknown. Medicinal plants are unsuccessful in attaining the anticipated scale due to a shortage of scientific data on their safety and efficiency. Thus, systematic validation studies are required.

## Conclusion

Avicenna had a remarkable influence on the field of cardiology, and his role had the most prominent effects on the progress of cardiological science. In the third volume of the Canon of Medicine, Avicenna defined numerous cardiovascular conditions and disorders. HTN is among the most prevalent diseases in the world, though it can be regulated and prohibited, and causes many difficulties for affected patients. Many simple approaches can be adopted to regulate high BP, such as lifestyle changes, pharmacotherapy, or both.

## Future view

Traditional botanical research on medicinal plants suggests novel areas of study on the antihyper­tensive effects of medicinal plants. With regard to their safety and efficacy, medicinal plants can be processed to pro­duce natural medications; however, their effect should be confirmed by pharmacological research and clinical trials. Studies in the future, which will focus on elongated randomized trials, may be of assistance in clarifying the durable effects of medicinal plants. In addition, studies on different herbs with antihyper­tensive effects have been promising so far and will lead to the discovery of new antihyper­tensive herbal medicines in the near future.

## Ethical Issues

Not applicable.

## Conflict of Interest

Authors declare no conflict of interest.

## Acknowledgments

Authors would like to acknowledge Department of Persian Medicine, Faculty of Traditional Medicine, Tabriz University of Medical Sciences, Tabriz, Iran for their great help.
